# Gastrointestinal parasite infections and self-medication in wild chimpanzees surviving in degraded forest fragments within an agricultural landscape mosaic in Uganda

**DOI:** 10.1371/journal.pone.0180431

**Published:** 2017-07-10

**Authors:** Matthew R. McLennan, Hideo Hasegawa, Massimo Bardi, Michael A. Huffman

**Affiliations:** 1 Anthropology Centre for Conservation, Environment and Development, Oxford Brookes University, Oxford, United Kingdom; 2 Bulindi Chimpanzee and Community Project, Hoima, Uganda; 3 Department of Infectious Disease Control, Faculty of Medicine, Oita University, Hasama, Yufu, Oita, Japan; 4 Department of Biology, Faculty of Medicine, Oita University, Hasama, Yufu, Oita, Japan; 5 Department of Psychology and Behavioral Neuroscience, Randolph-Macon College, Ashland, Virginia, United States of America; 6 Primate Research Institute, Kyoto University, Inuyama, Japan; University of Florida, UNITED STATES

## Abstract

Monitoring health in wild great apes is integral to their conservation and is especially important where they share habitats with humans, given the potential for zoonotic pathogen exchange. We studied the intestinal parasites of wild chimpanzees (*Pan troglodytes schweinfurthii*) inhabiting degraded forest fragments amid farmland and villages in Bulindi, Uganda. We first identified protozoan and helminth parasites infecting this population. Sixteen taxa were demonstrated microscopically (9 protozoa, 5 nematodes, 1 cestode, and 1 trematode). DNA sequence analysis enabled more precise identification of larval nematodes (e.g. *Oesophagostomum stephanostomum*, *O*. *bifurcum*, *Strongyloides fuelleborni*, *Necator* sp. Type II) and tapeworm proglottids (genus *Bertiella*). To better understand the ecology of infections, we used multidimensional scaling analysis to reveal general patterns of association among parasites, climate, and whole leaf swallowing–a prevalent self-medicative behaviour at Bulindi linked to control of nodular worms (*Oesophagostomum* spp.). Prevalence of parasites varied with climate in diverse ways. For example, *Oesophagostomum* sp. was detected in faeces at higher frequencies with increasing rainfall but was most clearly associated with periods of low temperature. Certain parasites occurred together within chimpanzee hosts more or less frequently than expected by chance. For example, the commensal ciliate *Troglodytella abrassarti* was negatively associated with *Balantidium coli* and *Oesophagostomum* sp., possibly because the latter taxa make the large intestine less suitable for *T*. *abrassarti*. Whole leaves in faeces showed independent associations with the prevalence of *Oesophagostomum* sp., *Strongyloides* sp., and hookworm by microscopic examination, and with egestion of adult *O*. *stephanostomum* by macroscopic inspection. All parasites identified to species or genus have been reported in wild chimpanzees inhabiting less-disturbed environments than Bulindi. Nevertheless, several disease-causing taxa infecting these chimpanzees are potentially transmissible between apes and humans (e.g. rhabditoid and strongyle nematodes), underscoring the importance of identifying and reducing risks of pathogen exchange in shared landscapes.

## Introduction

Non-human primates worldwide are increasingly found in modified and degraded habitats in proximity to humans and their domestic animals [1–3]. Understanding how anthropogenic habitat disturbance including farming, fragmentation and logging influence the gastrointestinal parasite faunas of wild primates consequently has strong relevance for conservation [4,5], with some studies reporting general links between disturbance and changes to host–parasite dynamics of primates (e.g. 6–10]; but see [11,12]). At the same time, growing contact between people and non-human primates (hereafter referred to as ‘primates’) calls for improved understanding of the risks of zoonotic pathogen transmission in shared habitats (e.g. [13–18]).

Like many primates, great apes are severely threatened by habitat loss for agriculture [19]. Unless heavily hunted or persecuted, however, populations of great apes can survive in degraded forests, plantation landscapes and forest–farm ecotones near human settlements [20–23]. The close phylogenetic relationship between humans and great apes results in a high potential for pathogen exchange in such habitats, raising concerns over public health [24–26] and great ape conservation [27]. Therefore, parasitological surveys of apes sharing habitats with people are useful for understanding the possible consequences of close human–ape coexistence by identifying taxa with pathogenic and zoonotic potential (e.g. [24,28–32]).

Parasitological surveys of wild chimpanzees (*Pan troglodytes*) have been made in various habitats across tropical Africa including lowland rainforest [33,34], mid-altitude forest [35–38], forest–woodland [39–41] and savanna-woodland [42–45], mostly at sites with low to moderate human disturbance. The few studies explicitly made in more disturbed habitats, e.g. forest fragments where spatial overlap and interactions with local humans is high, report parasite faunas which are overall comparable to other chimpanzee populations [30,46]. However, Sá et al. [30] found the highest prevalence of *Giardia intestinalis* in chimpanzees inhabiting fragmented parts of Cantanhez National Park, Guinea-Bissau, which they attributed to greater contact with humans and livestock. Cibot et al. [32] compared two chimpanzee groups in Kibale National Park, Uganda, and found that those with a home range more heavily impacted by human activities had a higher prevalence and load of hookworm-like eggs.

Many common parasites of chimpanzees are non-pathogenic (e.g. commensal protozoa which aid digestion [47,48]) or have unclear clinical significance, but several have strong pathogenic potential. For example, heavy infections of nodular worms (*Oesophagostomum* spp.) are associated with morbidity and mortality in some chimpanzee populations (i.e. Mahale [39] and Gombe [49], both in Tanzania). These strongyle nematodes are transmissible among humans, chimpanzees and other sympatric primates in some environments (e.g. Kibale National Park, Uganda: [16,32]). Additionally, *O*. *stephanostomum* specifically has been shown to be the primary target of medicinal plant use (bitter pith chewing and rough leaf swallowing) by chimpanzees at Mahale [39,50–52] There, bitter pith chewing resulted in the rapid decline in *O*. *stephanostomum* infection intensity (eggs per gram faeces) within 24 hours [51]. Swallowing rough or bristly leaves increases gut motility causing expulsion of adult worms, which disrupts the nematode’s life cycle and likely reduces worm burdens [53]. Thus, whole undigested leaves occurred in association with adult *Oesophagsotomum* in chimpanzee faeces at Mahale [52] and also at Bulindi, Uganda [54]. A similar relationship between leaf swallowing and tapeworm (*Bertiella* sp.) infection was reported from Kibale [55] and Budongo, Uganda [56], but was not apparent at Bulindi [54].

Temporal or seasonal variation in infections occur, reflecting climatic influences on parasite life cycles, changes in host diet or habitat use, and/or methodological differences among studies for observable effects [36,39,40,56,57]. At Mahale, for example, *Oesophagostomum* infection intensity increases rapidly following the onset of the wet season, during which leaf swallowing occurs most frequently [39]. Co-infection of chimpanzee hosts by multiple parasites, measured as species richness, is routinely observed in wild populations (e.g. [37,40,45,52]). To date, few studies have considered patterns of association among parasites of chimpanzees, including specific within-host associations, although such interactions are well-established in other species [58–60]. In one study, Ebbert et al. [45] found that some pairs of taxa (e.g. *Trichuris* sp. and *Balantidium* sp.) occurred together in chimpanzee faeces at Mt. Assirik, Senegal, more often than expected by chance.

We examined the intestinal parasites of chimpanzees in Bulindi, Uganda, using coproscopic and molecular methods. In Bulindi, chimpanzees inhabit small degraded forest fragments on agricultural land in remarkably close contact with humans and domestic animals [61–63]. An earlier study reported an unusually high frequency of whole leaf swallowing among Bulindi chimpanzees compared to reported frequencies elsewhere [54], potentially suggesting heightened vulnerability to certain pathogens. Moreover, a relationship between egestion of adult *Oesophagostomum* sp. and the presence of whole leaves in the chimpanzees’ faeces was found [54]. However, data from microscopic and molecular analysis were not available at that time. Thus, in the present study we:

1. Identified gastrointestinal parasites (protozoa and helminths) infecting Bulindi chimpanzees via coproscopic examination and molecular analysis of nematode larvae and macroscopic parasites.
2. Evaluated climatic influences on temporal patterns of parasite infections at Bulindi.
3. Examined temporal associations among parasites infecting the chimpanzee community, as well as associations of parasites within chimpanzee hosts.
4. Evaluated the relationship between leaf swallowing and infections with potentially pathogenic parasites including *Oesophagostomum*, assayed by both microscopic and macroscopic examination.
5. Identified parasites infecting the chimpanzees that have zoonotic and pathogenic potential to assess public health risk in this location of markedly close human–great ape contact.

## Materials and methods

### Study site

**Fig 1:**
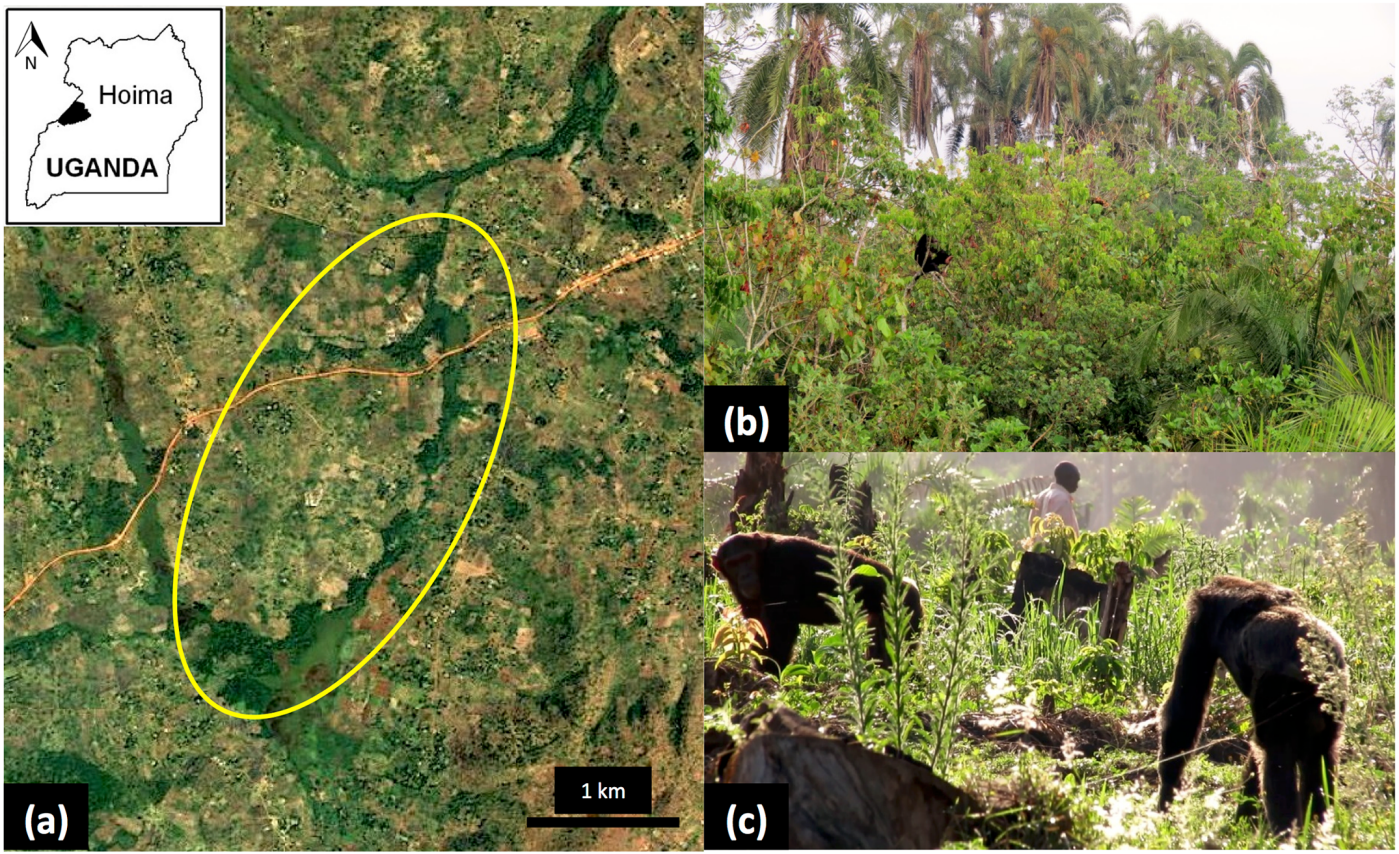
Home range and habitat of chimpanzees in Bulindi, Hoima District, Uganda. **(a)** Map adapted from Landsat imagery courtesy of USGS/NASA Landsat. The most commonly used portion of the home range is indicated by the yellow oval. Dark green areas are fragments of riverine forest, *Cyperus papyrus* swamp, regenerating bush and woodland; the surrounding matrix comprises smallholder farmland, homes and trading centres. The thin line at centre is a main road connecting Hoima and Masindi towns. **(b)** Typical view of narrow riverine forest dominated by tall *Phoenix reclinata* palms; a chimpanzee is visible in a small tree above the water. **(c)** Two adult males of the Bulindi community travelling across farmland while a local farmer (visible between the two chimpanzees) tends to his garden.

Bulindi (1°29′N, 31°28′E) is located in western Uganda’s Hoima District. Chimpanzees at Bulindi are among multiple chimpanzee groups (‘communities’) inhabiting degraded forest fragments on farmland across an area exceeding 600 km^2^ [61,64–66]. In 2014 the human population density in Hoima District exceeded 150 persons per km^2^ [67]. During 2012–2013, when this survey was made, the Bulindi chimpanzee community comprised 19 individuals: 6 adult females, 3 adult males, a subadult female and subadult male, and 8 juveniles and infants. Their home range (c. 20 km^2^) comprises narrow fragments of riverine forest amid agricultural gardens and village settlements, and is dissected by a main road (Fig 1). Common trees in fragments include the palm *Phoenix reclinata*, *Pseudospondias microcarpa*, *Funtumia africana*, and members of the Moraceae family, including *Trilepisium madagascariensis*, *Antiaris toxicaria* and figs *Ficus* spp. [68]. Chimpanzees in Bulindi are sympatric with black and white colobus monkeys (*Colobus guereza*), tantalus monkeys (*Chlorocebus tantalus*), and olive baboons (*Papio anubis*). Solitary or small groups of blue monkeys (*Cercopithecus mitis*) and red-tailed monkeys (*Cercopithecus ascanius*) have occasionally been seen.

Rainfall in the region is bimodal. March–May and August–November are usually wet (>100 mm rainfall monthly), December–February are dry (≤50 mm rainfall monthly), while June–July are ‘transient’ (51–100 mm rainfall), following the classification scheme used by Newton-Fisher [69] and Huffman et al. [56] for western Uganda. However, some inter-annual variation in rainfall distribution occurs. Highest maximum and lowest minimum temperatures generally occur in January–March.

This survey was undertaken concurrent with major habitat disturbance in Bulindi: beginning c.2000 all forest within the chimpanzees’ range was logged [63,68] and most forest was converted to farmland (c. 80% loss during 2006–2014). The chimpanzees frequently enter gardens to feed on agricultural crops [70,71] and they encounter villagers and domestic animals daily (including cattle, chickens, pigs, goats, dogs and cats) [62] (Fig 1). Villagers sometimes defecate outdoors at garden edges and in the forest, and chimpanzee knuckle prints have been seen in fresh human faeces [54]. Conversely, chimpanzees defecate in gardens and by homes when travelling and foraging between forest fragments. Villagers collect water from streams within forest fragments. Thus, there is a risk of mutual parasite transmission in Bulindi.

### Sample collection

Chimpanzee faecal samples were collected in two survey periods: September–November 2012 (period 1) and February–April 2013 (period 2). Samples were collected non-invasively in the first half of the day (between 0700–1300) from fresh faeces deposited that morning. While chimpanzees were individually identifiable, they were not habituated to close observation. Thus, samples were collected from anonymous individuals at recently vacated nest sites and during tracking. The Bulindi community is small and cohesive, and individuals presumably had an equal chance of being sampled each day. In total, 432 samples were collected for microscopic examination. At collection, c.1 g faeces was placed in 5 mL tubes prefilled with 10% formalin solution and shaken vigorously.

Additionally, 406 faeces were inspected macroscopically for proglottids and adult nematodes, and undigested whole leaves indicative of self-medication (period 1 = 164; period 2 = 242), including 362 for which a sample was also collected for coproscopic analysis. The discrepancy in number of faeces examined microscopically and macroscopically is because some faeces collected for coproscopy were too small for visual inspection (e.g. when only small pieces of faeces were recovered below a tree), while some faeces were judged potentially older than 6 h (our limit for coproscopic analysis) and were collected for visual inspection only. All macroscopic parasites were fixed in ≥99% ethanol.

In April 2013, 38 faecal samples were subjected to the modified Harada–Mori filter paper culture [72], irrespective of presence of adult worms. After 7–14 days, larva-positive water was transferred to a 5 mL tube and fixed in ≥99% ethanol [73]. All samples were transported to the Department of Biology, Faculty of Medicine, Oita University, Japan for analyses.

### Laboratory analysis

Fifty samples were examined initially via simple sedimentation and formalin–ether sedimentation methods [74]. In the former method, processing of faeces was the same as in formalin–ether sedimentation except sedimentation was done without adding ether, after which the supernatant was discarded and the sediment was spread under the cover slip and examined. Simple sedimentation performed marginally better (mean species richness was 3.4 by simple sedimentation versus 3.2 by formalin–ether). Thereafter samples were examined solely via simple sedimentation and only results from this method are reported. The gauze used for straining faeces was inspected for worm bodies such as pinworms (*Enterobius* sp.) following Hasegawa [72]. Microscopic identification was made with reference to Hasegawa et al. [75] and Modrý et al. [76].

DNA sequence analysis was performed for (i) tapeworm proglottids and adult nematodes found in faeces, and (ii) nematode larvae from coprocultures. From each proglottid, a small fragment (about 1 mm^3^) was resected using a sterilized disposable scalpel blade, and washed in sterilized distilled water. Then, the fragment was homogenised in 100 μL of sterilised distilled water in a sterilised Eppendorf tube using a sterilised pestle. Five μL of the solution was mixed with 50 μL of the liquid phase of Dexpat (Takara Bio. Inc., Otsu, Shiga, Japan) in a 200 μL PCR tube, heated at 100°C for 20 min, and cooled on ice to prepare a template solution. Subsequently, 15 μL of the template solution was added to the 60 μL PCR mixture containing 1 μL of KOD-Neo polymerase (Toyobo Co., Tokyo, Japan). PCR was performed using a thermal cycler (PC-801, ASTEC Co., Ltd., Fukuoka, Japan). The primer sets used for amplification of ITS1 and ITS2 were BerITS1-F (forward: 5’-CTGCGGAAGGATCATTACAC-3’) and BerITS1-R2 (reverse: 5’-GCAGTCTGCGATTCACATTA-3’), and BerITS2-F2 (forward: 5’-TAATGTGAATCGCAGACTGC-3’) and BerITS2-R2 (reverse: 5’-CGCCGTTACCAAGGGAATC-3’), respectively. Methods for DNA extraction and PCR for nematodes were described previously [73,77].

PCR was carried out with an initial denaturation at 94°C for 2 min, followed by 20 cycles of 98°C for 10 sec, 50°C for 1 min, 68°C for 1 min, 20 cycles of 94°C for 1 min, 55°C for 1 min, 68°C for 1 min, and a post-amplification extension at 68°C for 7 min. The PCR products were mixed with EzVision Three DNA Dye (Amresco, Solon, Ohio, USA) and electrophoresed in a 1.5% agarose gel. Following electrophoresis, DNA bands were extracted from the gel and purified with SUPREC-01 column (Takara Bio Inc.). Products were then subjected to direct sequencing using the BigDyeTerminator cycle sequencing kit v. 3.1 (Applied Biosystems, Foster City, California, USA), and an automated genetic analyzer ABI-PRISM 3130 (Applied Biosystems). DNA sequences obtained were registered in DDBJ (DNA Data Bank of Japan)/EMBL-Bank/GenBank (accession numbers: LC185219–LC185221, LC185224–LC185229).

Molecular features of nematodes in the genera *Oesophagostomum*, *Strongyloides* and *Necator* from Bulindi chimpanzees are reported elsewhere [73,78,79]. Here, additional molecular analysis of larvae from coprocultures and proglottids recovered from their faeces is presented. The programs FASTA version 36.3.7b [80] at EBI (European Bioinformatics Institute) and BLAST version 2.2.26 [81] at DDBJ were used to identify sequences with high similarity to DNA sequences obtained from the proglottids and larvae.

### Data analysis

The sample prevalence (proportion or frequency of faecal samples positive for a given parasite taxa) was used as a proxy for individual prevalence. Because we did not measure the intensity of samples (eggs per gram faeces) and chimpanzee’s identity of each sample was unknown, our analysis is intended to reveal only general patterns of parasite infection in the Bulindi community. Since individual chimpanzees contributed multiple samples, non-independency might have been a problem in our dataset. We therefore ran autocorrelation function (ACF) tests to detect autocorrelation and partial correlation in all parasite taxa detected microscopically. AFC tests return the correlation coefficients between two values (faecal samples) of the same variable (any given parasite) at times *X*_*i*_ and *X*_*i+k*_. Given measurements, *Y*_1_, *Y*_2_,…, *Y*_*N*_ at time *X*_1_, *X*_2_,…, *X*_*N*_, the lag *k* autocorrelation function is defined as:

$$r_{k}=\sum_{i=1}^{n-k}(y_{k+i}-\bar{y})(y_{i}-\bar{y})/\sum_{i=1}^{n}(y_{i}-\bar{y}{)}^{\begin{array}{l} 2 \end{array}}$$

Although the time variable, *X*, is not used in the formula for autocorrelation, the assumption is that the observations are equally spaced. When the autocorrelation is used to detect non-randomness, only the first few lag autocorrelations are usually of interest [82]. Only a single point of significant autocorrelation (p<0.05) was detected in the whole dataset (136 points in total), indicating that autocorrelation was not a concern in our dataset.

Chi-square tests were used to assess if our measure of prevalence differed (i) between survey periods and (ii) between faecal samples from months classified as ‘wet’ (n = 262) versus ‘dry’ or ‘transient’ months (n = 170); samples collected in dry or transient months were lumped because relatively few samples were available from February, the only dry month in the survey. We did not adjust for multiple tests since this initial chi-square analysis was exploratory and p-values were not used to make inferences about seasonality of infections (see below). In other analyses, survey months were divided into first and second halves, giving ten ‘biweekly periods’ (samples were collected in one half of the month only in September 2012 and February 2013). Parasite prevalence and climate variables (cumulative rainfall, mean relative humidity, and mean minimum and maximum temperature) were calculated for each biweekly period. Spearman rank correlations were used to assess associations between climate variables during August 2012–April 2013, i.e. 9 months beginning 1 month prior to period 1 through to the last month of period 2, giving 18 biweekly periods.

To examine seasonality of infections in more detail, we conducted a multidimensional scaling (MDS) analysis to provide a model of independent associations among the biweekly prevalence of different parasites and corresponding climatic conditions. Parasites selected for inclusion in the model were those that differed in prevalence between survey periods, as indicated by exploratory chi-square analysis, suggesting a possible influence of climate. Unlike some other multivariate techniques, MDS does not require linear and unimodal distributions of data (i.e. parasite taxa) along gradients—a condition not satisfied in our dataset. MDS is a data-reduction technique used to uncover a “hidden structure” to a set of data, which is not possible with standard parametric statistical techniques [83]. The MDS output returns a model that provides a spatial representation of the similarity among response variables (e.g. prevalence of individual parasites) and predictors (climatic variables). Using matrices built on Euclidean distances among variables, the proximities can be displayed in a two or higher dimensional space (i.e. a map of proximities). Within the map, the closer two or more variables are, the more likely they covary independently of other points on the map. Conversely, the farther apart they are, the less likely the predictors (e.g. climate variables) can explain the response variable (parasite prevalence). The underlying dimensions are thought to “explain” the perceived similarity among variables. Since this function is often conceived of as a weighted sum of the similarity across individual variables, where the weights reflect the importance (saliency) and direction (negative or positive weights) of the variable, the location of variables in the map depend on these weights. In other words, close similarities can be attributed to both positive and negative covariations among variables.

This approach has proven particularly useful in studies of ecological diversity and species prevalence [84,85]. In order to map all variables into a desired space (two dimensional or greater), a certain lack of fit—i.e. uncertainty around points in the map—has to be accepted. This lack of fit is referred to as “s-stress’”. The values of s-stress range from 0 (perfect fit) to 1 (worst possible fit). The aim of MDS is to find a map of the variables that minimizes the s-stress for a given number of dimensions. Stress values below 0.15 (15%) are typically deemed acceptable. At the same time, MDS attempts to maximize the variance explained by the model (R^2^), as is the case with any regression analysis. Typically, R^2^ values of 0.8 (80%) or higher are desirable. Finally, one must pick a solution based on interpretability of the dimensions. Parsimony is crucial to interpreting the associations among predictors and response variables. The number of dimensions can be likened to the number of latent underlying factors in the dataset. Thus, when choosing the number of dimensions to represent the data, one must consider (i) the number of variables in the model, (ii) the lack of fit (s-stress value) given the number of dimensions, (iii) an index of fit of the model (R^2^), and (iv) interpretability of the dimensions.

A second MDS model was used to reveal the reciprocal, independent associations among parasites most commonly detected in faecal samples (>5% of samples) in biweekly periods. We also ran chi-square tests to identify pairs of parasites that occurred together *within* chimpanzee hosts (i.e. within the same faecal samples) more or less often than expected by chance. When an expected cell frequency was <5, we used Fisher’s exact test instead. To control for multiple tests we applied a Holm–Bonferroni sequential adjustment to the P-values [86].

A third MDS model was used to reveal the independent associations among the frequency of whole leaves in faeces and prevalence of six parasites of known or likely pathogenicity, including *Oesophagostomum* sp. Rainfall was also entered into the model since leaf swallowing occurred most frequently during wet seasons at some sites [39,87]. Because samples were not identified to individual chimpanzees we were unable to test specific relationships between leaf swallowing and parasite infections within individuals. We used Fisher’s exact test to determine if whole leaves and egested adult *Oesophagostomum* worms occurred together in faecal samples more often than expected. The analysis was performed using SPSS Version 23 (SPSS Inc., Chicago, IL, USA). All probabilities reported are two-tailed.

### Ethics statement

This research involving wild chimpanzees was entirely non-invasive and adhered strictly to the legal requirements of Uganda, and to ethics guidelines detailed by the Association for the Study of Animal Behaviour (UK) and the American Society of Primatologists Principles for the Ethical Treatment of Nonhuman Primates. Ethical approval was granted by the Uganda National Council for Science and Technology, the President’s Office, the Uganda Wildlife Authority, and Oxford Brookes University Research Ethics Committee.

## Results

### Parasite diversity and species identification

**Fig 2:**
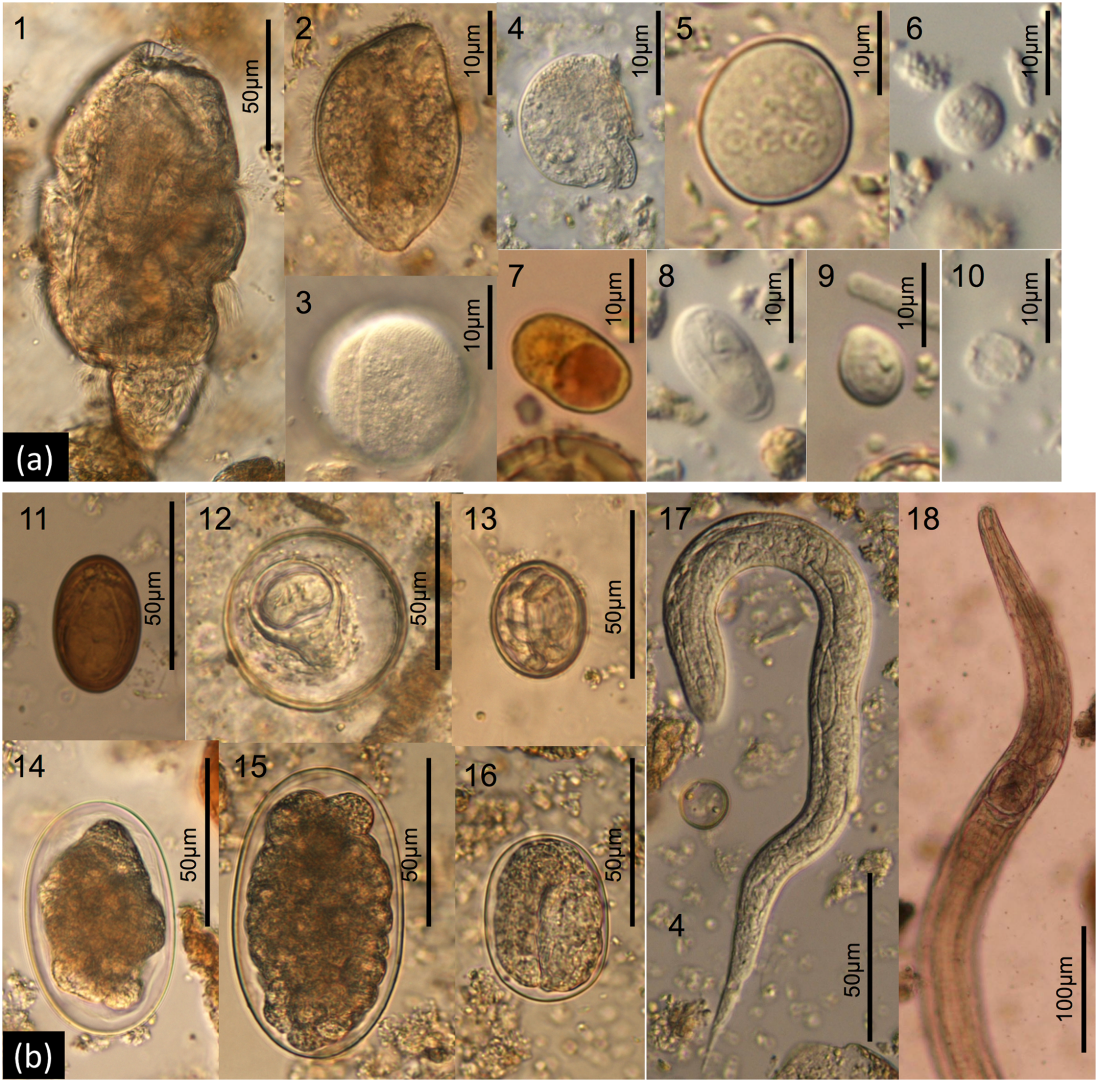
Gastrointestinal parasites and symbionts found from coproscopic analysis of faeces of chimpanzees in Bulindi, Uganda. **(a)** Protozoa: 1. *Troglodytella abrassarti*; 2. *Balantidium coli* (trophozoite); 3. *Balantidium coli* (cyst); 4. *Troglocorys cava*; 5. *Entamoeba coli*; 6. *Entamoeba* sp.; 7. *Iodamoeba buetschlii*; 8. *Giardia intestinalis*; 9. *Chilomastix mesnili*; 10. *Blastocystis* sp. **(b)** Helminths: 11. Dicrocoeliidae gen. sp.; 12. *Bertiella* sp.; 13. Spiruridae gen. sp.; 14. Hookworm; 15. *Oesophagostomum* sp.; 16. *Strongyloides* sp. (egg); 17. *Strongyloides* sp. (larva); 18. *Probstmayria gombensis* (adult).

At least 16 distinct parasite taxa were identified by coproscopy (Table 1) including 9 protozoa (*Balantidium coli*, *Blastocystis* sp., *Chilomastix mesnili*, *Entamoeba coli*, *Entamoeba* sp., *Giardia intestinalis*, *Iodamoeba buetschlii*, *Troglocorys cava*, *Troglodytella abrassarti*; Fig 2a), 5 nematodes (hookworm, *Oesophagostomum* sp., *Probstmayria gombensis*, *Strongyloides* sp., Spirurida fam. gen. sp.), 1 cestode (*Bertiella* sp.) and 1 trematode (Dicrocoeliidae gen. sp.) (Fig 2b). Cysts assigned to *Entamoeba* sp. potentially comprised plural *Entamoeba* species with morphologically indistinguishable cysts [88] and also included cysts resembling *Endolimax nana* by having light reflective nuclei. Pinworms (*Enterobius* sp.) were not observed in the samples.

**Table 1:** Prevalence of microscopic and macroscopic gastrointestinal parasite taxa in faeces of chimpanzees in Bulindi, Uganda, during the two survey periods (1 = Sep–Nov 2012; 2 = Feb–Apr 2013).

	Survey period and season			
Parasite taxa	Overall	1 (Wet)	2 (Transitional: dry to wet)	
(1) Parasite taxa identified from coproscopy ^a^	**Prevalence (% samples)**			
Protozoa				
*Balantidium coli* (cyst & trophozoite)	5.1	5.8	4.6	
*Blastocystis* sp. (cyst)	7.4	0.6	11.9	***
*Chilomastix mesnili* (cyst)	0.2	0.6	0	
*Entamoeba coli* (cyst)	8.3	2.3	12.3	***
*Entamoeba* sp. or spp. (cyst) ^b^	39.1	40.1	38.5	
*Giardia intestinalis* (cyst)	1.6	0.6	2.3	
*Iodamoeba buetschlii* (cyst)	3.0	2.3	3.5	
*Troglocorys cava* (trophozoite)	15.3	11.0	18.1	*
*Troglodytella abrassarti* (trophozoite)	79.6	78.5	80.4	
Nematoda				
Hookworm (egg) ^c^	44.2	43.0	45.0	
*Oesophagostomum* sp. (egg) ^d^	48.1	54.7	43.8	*
*Probstmayria gombensis* (adult)	16.0	5.8	22.7	***
*Strongyloides* sp. (egg & larva)	57.9	50.6	62.7	*
Spirurida fam. gen. sp. (egg)	0.2	0	0.4	
Free-living nematodes (adult & larva) ^e^	23.1	24.4	22.3	
Cestoda				
*Bertiella* sp. (egg)	0.7	0.6	0.8	
Trematoda				
Dicrocoeliidae gen. sp. (egg)	0.2	0	0.4	
(2) Macroscopic parasites identified from DNA sequence analysis ^f^	**% faeces with macroscopic parasite**			
*Bertiella* sp. (proglottid) ^g^	0.7	0	1.2	
*Oesophagostomum stephanostomum* (adult) ^h^	2.5	1.8	2.9	

Chi-square tests for differences in prevalence between survey periods:

* P<0.05

*** P<0.001

other comparisons were P>0.05

^a^ No. faecal samples examined by coproscopy (N = 432): period 1 = 172; period 2 = 260.

^b^ Some cysts assigned to *Entamoeba* sp. had nuclei resembling *Endolimax nana*. However, it was not possible to differentiate these conclusively from morphological characters alone.

^c^ Presumably *Necator* sp. A small proportion of eggs assigned to ‘hookworm’ may include *Oesophagostomum bifurcum* eggs, which are very similar to those of hookworm.

^d^ Presumably *O*. *stephanostomum*. Although *O*. *bifurcum* was also identified by molecular analysis (Table 2), the eggs are smaller and not easily distinguished from those of hookworm by microscopy. A small proportion of eggs assigned to *Oesophagostomum* sp. may have included trichostrongyle eggs, which are very similar to those of *Oesophagostomum* sp.

^e^ Small free-living nematodes potentially resulting from post-defecation contamination belonged to plural (unidentified) species. These were excluded from species richness calculations.

^f^ No. faeces inspected for macroscopic parasites (N = 406): period 1 = 164; period 2 = 242.

^g^ A single proglottid was observed in 3 different faeces during Period 2; each was assigned to ‘*Bertiella* sp.’ (see text).

^h^ 17 adults from 10 different faeces were each assigned to *O*. *stephanostomum* [73].

One or more parasite taxa were detected in 99% of the samples examined microscopically. Mean species richness (number of different taxa per sample) was 3.3 (range 0–9).

DNA sequence analysis enabled more precise identification of nematodes. As reported previously (see Table 2), larval *Oesophagostomum* were assigned to *O*. *stephanostomum* and *O*. *bifurcum*, with the former being predominant. All adult worms observed macroscopically were *O*. *stephanostomum* (Table 1). *Strongyloides* larvae were assigned to *S*. *fuelleborni*, and hookworm larvae were assigned to ‘*Necator* sp.’–probably *N*. *gorillae*. A trichostrongyloid with filariform larva comparable in size to that of *Necator* sp. was additionally demonstrated in four cultures by agarose gel electrophoresis of PCR products (Table 2). Sequencing was attempted for five larvae, and a sequence with 506 base pairs (bp) covering partial ITS1 (111 bp), whole 5.8S rDNA (153 bp), and whole ITS2 (242 bp) was obtained (accession numbers LC185219 to LC185221). While some undetermined nucleotides were scattered in the sequences, a consensus sequence (506 bp) could be formed. By both FASTA and BLAST similarity analyses, it aligned closest to *Libyostrongylus douglassi* (sequence identities 93–94%), followed by *Libyostrongylus dentatus* (identity 92%).

**Table 2:** Summary of DNA sequence analysis of larval nematodes from coprocultures (n = 38).

Nematode larvae	No. (%) cultures positive	No. larva analysed	Species identification	Reference
*Oesophagostomum* spp.	22 (58%)	15	1. *O*. *stephanostomum* 2. *O*. *bifurcum*	[73]
*Strongyloides* sp.	24 (63%)	14	*S*. *fuelleborni*	[78]
Hookworm	25 (66%)	34	*Necator* sp. (Type II)	[79]
Trichostrongyloid	4 (11%)	5	Trichostrongyloidea fam. gen. sp. (?*Libyostrongylus* sp.)	This study

DNA sequence analysis was performed on three proglottids observed macroscopically in three different faecal samples (labelled ‘D421’, ‘D422’, and ‘D455’, respectively). The partial ITS1 sequences of two proglottids were composed of 652 bp (LC185227, LC185228), while 608 bp lacking 44 bp at 5’ side of the longer sequence was sequenced in the remaining one (LC185229). Only one substitution (T with A) was found at the 30th position of the longer sequences. The full length of ITS2 with 638 bp was successfully sequenced for one proglottid (D455) (LC185224). For the other two, sequences were apparently the same in length with that of D455 although 3 and 65 nucleotides remained undetermined for the D422 (LC185225) and D421 (LC185226) proglottids, respectively. Between the D455 and D422 proglottids, a nucleotide substitution (T with A) was observed at the 195th and 318th positions. By FASTA similarity analysis, the ITS1 sequence aligned closest to *Bertiella studeri* sequences from two humans in Japan (AB586127, AB586128) (identities 79% in 563 nucleotides), followed by *Bertiella satyri* from a Sumatran orangutan (*Pongo abelii*) (EU908290) (85% in 341 nucleotides). Conversely, by BLAST analysis, the ITS1 sequence aligned closest to *B*. *satyri* from the Sumatran orangutan, followed by the two sequences of *B*. *studeri* from Japanese humans. By FASTA analysis, the ITS2 sequence of the D455 proglottid aligned closest to *Bertiella* sp. from a Ugandan chimpanzee (JQ771097) (identity 99% in 619 nucleotides), followed by *Anoplocephala* cf. *gorillae* from *Gorilla beringei* in Rwanda (JQ771100) (97% in 629 nucleotides), *Bertiella* sp. from *Gorilla gorilla* of Central African Republic (JQ771099) and *B*. *mucronata* from *Callicebus oenanthe* of Peru (JQ771096) (90% in 619 nucleotides in both cases), *B*. *studeri* from a Spanish human (JQ771094) (79% in 482 nucleotides), *Bertiella* sp. from a Brazilian human (JQ771093) and *B*. *satyri* from an Indonesian orangutan (JQ771195) (76% in 375 nucleotides in both cases), *B*. *studeri* from the two Japanese humans (AB586129, AB586130) (73% in 309 nucleotides), and *Bertiella* sp. from a sanctuary chimpanzee in Kenya (72% in 365 nucleotides). A similarity search of the ITS2 sequence by BLAST analysis gave almost the same order of homology to that found by FASTA analysis.

### Parasite prevalence

The highest prevalence was found for an entodiniomorphid ciliate *Troglodytella abrassarti* (80% of samples examined microscopically) (Table 1). Three pathogenic nematodes also occurred at a relatively high prevalence: eggs and/or larvae of *Strongyloides* sp. (58%), and eggs of *Oesophagostomum* sp. (48%) and hookworm (44%). Cysts assigned to ‘*Entamoeba* sp.’ were detected in 39% of all samples. All other parasites were detected at a low prevalence by coproscopy (<20% samples) including cysts of potentially pathogenic protozoa *Balantidium coli* and *Giardia intestinalis*, and *Bertiella* sp. eggs. Three taxa were detected in only one sample (0.2%): eggs of a *Dicrocoelium*-like fluke and a Spirurid-like nematode, and cysts of a flagellate *Chilomastix mesnili*. Several parasites were detected at a notably higher frequency in one of the two survey periods (Table 1).

### Seasonality

**Fig 3:**
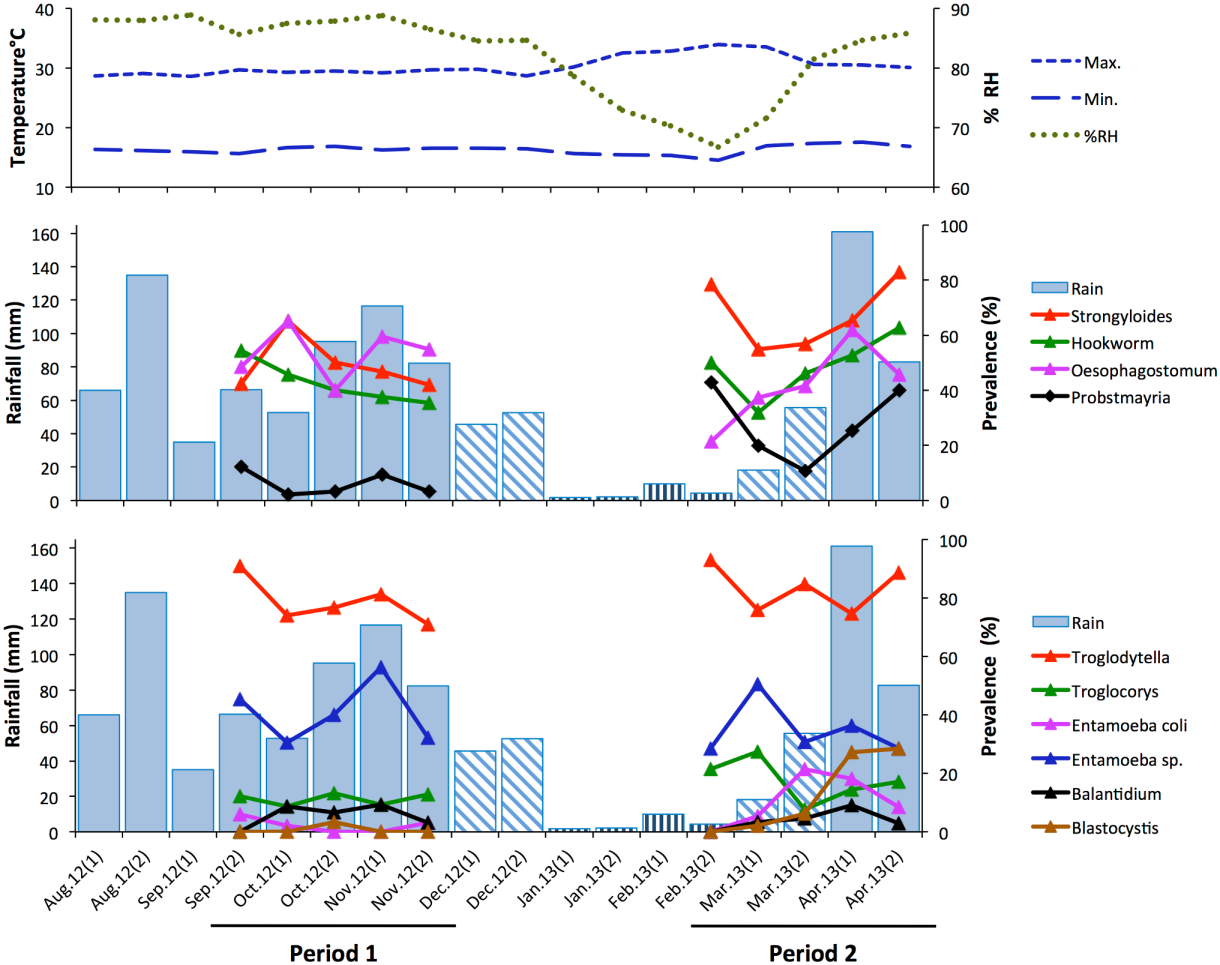
Variation in parasite prevalence in relation to rainfall, temperature and humidity. Climate data are shown for the period August 2012–April 2013, spanning both survey periods and beginning one month prior to period 1. **Top**: Biweekly mean daily maximum and minimum temperature and % relative humidity (RH); **Middle**: Biweekly rainfall and % faecal samples positive for nematodes (hookworm, *Oesophagostomum* sp., *Probstmayria gombensis* and *Strongyloides* sp.). **Bottom**: Biweekly rainfall and % faecal samples positive for protozoan parasites (*Balantidium coli*, *Blastocystis* sp., *Entamoeba coli*, *Entamoeba* sp., *Troglocorys cava*, *Troglodytella abrassarti*). The patterning of rainfall bars indicate whether biweekly periods were in months classified as wet (solid), transient (hatched) or dry (vertical lines); see Methods. Only parasites detected in >5% of samples (n = 432) are shown; for the number of faecal samples analysed per biweekly period, see Supporting Information S1 Data File.

The climate was more variable in survey period 2 compared to period 1 (Fig 3). Period 1 consisted of all wet months (September–November 2012) and was preceded by a wet month (August). In contrast, period 2 (February–April 2013) consisted of one dry (February), one ‘transient’ (March) and one wet (April) month, and was preceded by a dry month (January). Thus, period 2 was transitional between dry and wet seasons. Average daily temperatures and relative humidity were relatively constant throughout period 1. However, humidity dropped markedly in January (dry) through March (transient) when daily temperatures were hottest (Fig 3).

Mean daily maximum and minimum temperatures were not correlated across biweekly periods during August 2012–April 2013 (r_s_ = -0.037, n = 18, p = 0.88). However, mean maximum temperature was negatively correlated with mean relative humidity (r_s_ = -0.890, p <0.001), while rainfall correlated positively with mean relative humidity (r_s_ = 0.641, p = 0.004). Mean minimum temperature correlated positively with rainfall (r_s_ = 0.522, p = 0.026).

The prevalence of most parasites did not differ markedly between samples collected during wet months (September–November 2012 and April 2013) and dry or transient months (February–March 2013) (Table 3). However, *Blastocystis* sp. and particularly *Oesophagostomum* sp. were detected at a notably higher frequency in wet months.

**Table 3:** Parasite prevalence in wet months (>100 mm rainfall) and dry and transient months (≤100 mm rainfall) compared.

Parasite taxa ^a^	Prevalence (% samples)		
	Wet months	Dry–transient months	
Protozoa			
*Balantidium coli*	6.1%	3.5%	
*Blastocystis* sp.	9.9%	3.5%	*
*Entamoeba coli*	6.5%	11.2%	
*Entamoeba* sp. or spp.	37.8%	41.2%	
*Troglocorys cava*	12.6%	19.4%	
*Troglodytella abrassarti*	79.0%	80.6%	
Nematoda			
Hookworm	47.7%	38.8%	
*Oesophagostomum* sp.	55.0%	37.7%	***
*Probstmayria gombensis*	14.5%	18.2%	
*Strongyloides* sp.	58.0%	57.7%	

Chi-square tests for differences in prevalence between wet months (n = 262 faecal samples) and dry–transient months (n = 170):

* P<0.05

*** P<0.001

other comparisons were P>0.05.

^a^ Only parasites detected in >5% of faecal samples are shown.

**Fig 4:**
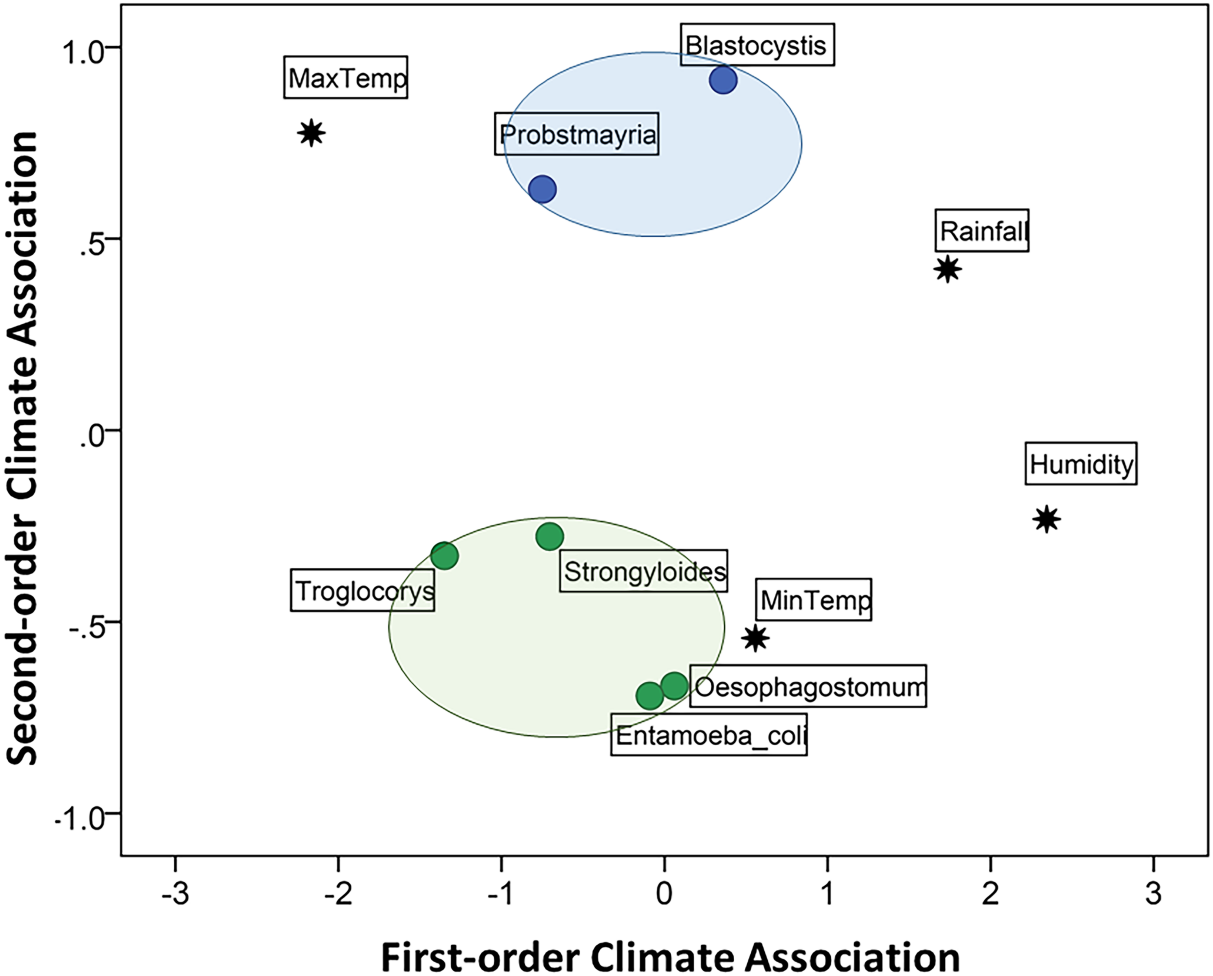
Multidimensional scaling (MDS) model of independent associations among parasites and climate. The first-order dimension represents a stronger association than the second-order dimension. The first dimension represents a combination of high rainfall and humidity (on the positive scores) and high temperature (negative scores) while the second represents a combination of high and low temperatures. The closer two or more variables are, the more likely they covary independently of other points on the map. Colours differentiate groups of variables that show some degree of association in relation to other variables in the map. Only parasite taxa that showed a significant difference in prevalence between survey periods were selected for the model (see Table 1).

Fig 3 plots biweekly parasite prevalence in relation to climate. The variables selected for the MDS model of independent associations among parasites and climate included the 4 climate predictors and the 6 parasite taxa that differed in prevalence between survey periods, as indicated by chi-square tests (see Table 1): *Blastocystis* sp., *Entamoeba coli*, *Troglocorys cava*, *Oesophagostomum* sp., *Probstmayria gombensis and Strongyloides* sp. The selection of these parasites from the 16 taxa identified by coproscopy was essential to satisfy the parsimony requirement of MDS for a two-dimensional output. The matrix of stimulus coordinates is presented in Fig 4; the matrix of optimal scaled distances is shown in Supporting Information S1 Table. The MDS returned a stress of 0.081 and an R^2^ = 0.967, indicating an excellent resolution of the hidden structure of the data for the selected variables. The good fit of the model showed an optimal R^2^ (0.908) for nonlinear fit in the data.

The first dimension of the MDS discriminated primarily between high (right quadrants) and low (left quadrants) rainfall and humidity (Fig 4). The second dimension discriminated primarily between high (the top two quadrants) and low (lower two quadrants) temperatures. MDS revealed a clear association between biweekly periods of low temperature and increased prevalence of *Oesophagostomum* sp. and *Entamoeba coli* in samples. *Troglocorys cava* and *Strongyloides* sp. were also more prevalent in periods of low temperature (although not as low as for *Oesophagostomum* sp. and *Entamoeba coli*) as well as low rainfall and low relative humidity. Finally, *Blastocystis* sp. and *Probstmayria gombensi*s were independently associated with high temperature, with increased prevalence during periods of higher precipitation and humidity for *Blastocystis* (as suggested by its position to the right on the first dimension) and lower rainfall and humidity for *Probstmayria* (positioned to the left on the first dimension) (Fig 4).

### Associations among parasites

**Fig 5:**
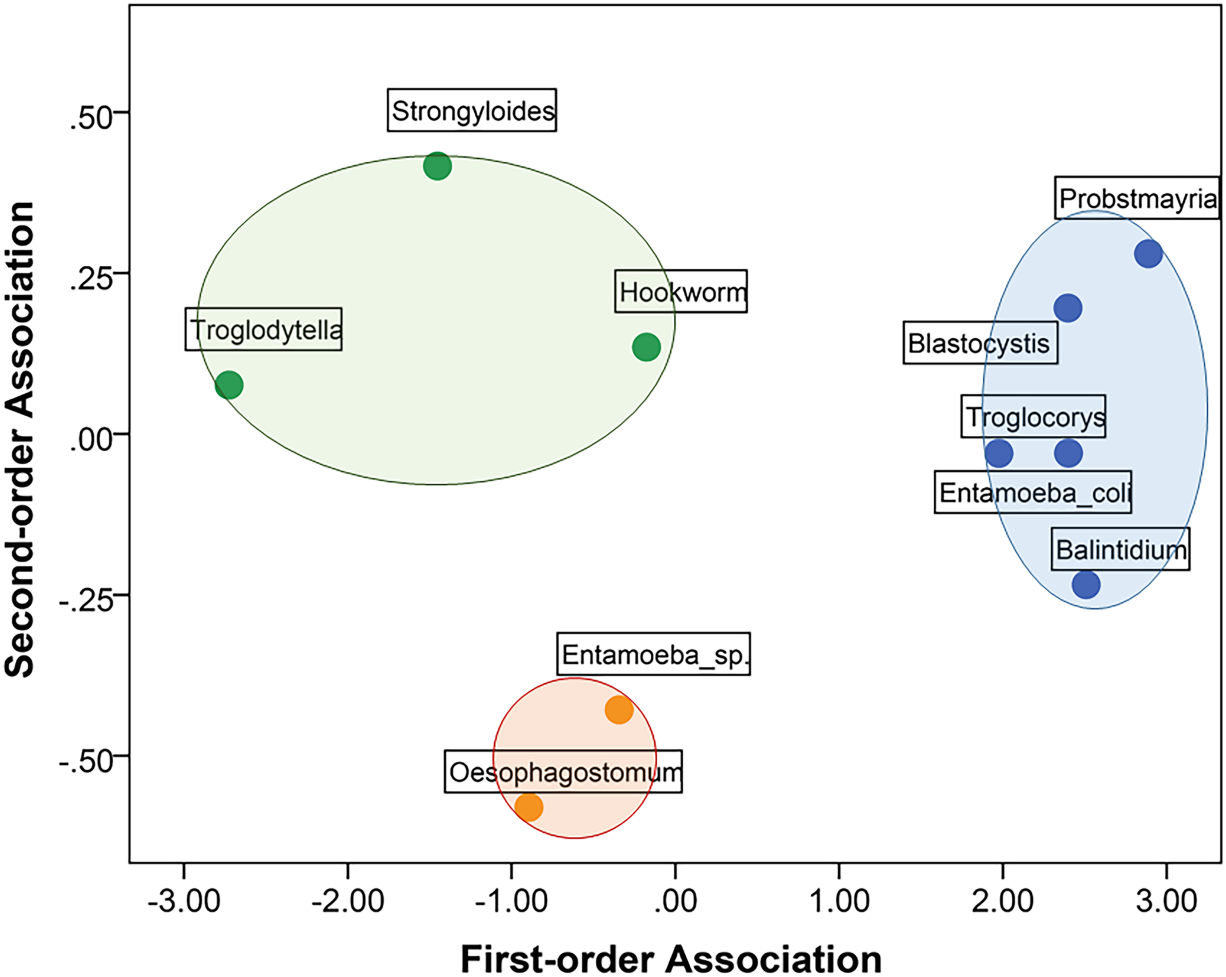
Multidimensional scaling (MDS) model of independent associations among parasites. The first-order dimension represents a stronger association than the second-order dimension. The closer two or more variables are, the more likely they covary independently of other points on the map. Colours differentiate groups of variables that show some degree of association in relation to other variables in the map. Only parasites detected in >5% of faecal samples were included in the model.

The second MDS model revealed three major clusters of parasites that showed temporal associations in prevalence (stress = 0.013 and R^2^ = 0.998) (Fig 5; the matrix of optimal scaled distances is shown in Supporting Information S2 Table). In the first cluster, on the right part of the diagram, *Blastocystis* sp. was associated with the nematode *Probstmayria gombensis* (with both taxa increasing in prevalence during the transitional period 2; Fig 3), and with three other protists: *Balantidium coli*, *Troglocorys cava* and *Entamoeba coli*. These taxa had similar overall prevalence (Table 1). In the second cluster, represented in the bottom part of the diagram, *Entamoeba* sp. and *Oesophagostomum* sp. showed an association. The final cluster, on the left side of the diagram, revealed a weak association among the commensal ciliate *Troglodytella abrassarti* and nematodes *Strongyloides* sp. and hookworm.

Several pairs of parasites occurred together *within* chimpanzee hosts (i.e. in the same faecal samples) significantly more or less often than expected (Table 4). The commensal ciliates *Troglodytella abrassarti* and *Troglocorys cava* were positively associated in samples. *Troglodytella*—the most commonly detected taxa—was negatively associated with two parasites with strong pathogenic potential: *Balantidium coli* and *Oesophagostomum* sp. Conversely, *Troglodytella* co-occurred in samples with *Strongyloides* sp. eggs and larvae more often than expected. *Strongyloides* sp. was negatively associated with *Balantidium coli* cysts and trophozoites, but had a positive association with *Blastocystis* sp. Two nematodes, hookworm and *Oesophagostomum* sp., showed a positive association within chimpanzee hosts.

**Table 4:** Within-host associations among parasites in chimpanzee faecal samples in Bulindi ^a^.

	*Balantidium coli*	*Blastocystis* sp.	*Entamoeba coli*	*Entamoeba* sp. or spp.	*Troglocorys cava*	*Troglodytella abrassarti*	Hookworm	*Oesophagostomum* spp.	*Probstmayria gombensis*	*Strongyloides* sp.
*Balantidium coli*	–									
*Blastocystis* sp.	n.a.	–								
*Entamoeba coli*	n.a.	n.a.	–							
*Entamoeba* sp. or spp.	n.a.	n.a.	n.a.	–						
*Troglocorys cava*	n.a.	n.a.	n.a.	n.a.	–					
*Troglodytella abrassarti*	**<0.00001 (-)**	n.a.	n.a.	n.a.	**<0.05 (+)**	–				
Hookworm	n.a.	n.a.	n.a.	n.a.	n.a.	n.a.	–			
*Oesophagostomum* sp.	n.a.	n.a.	n.a.	n.a.	n.a.	**<0.00001 (-)**	**<0.01 (+)**	–		
*Probstmayria gombensis*	n.a.	n.a.	n.a.	n.a.	n.a.	n.a.	n.a.	n.a.	–	
*Strongyloides* sp.	**<0.00001 (-)**	**<0.05 (+)**	n.a.	n.a.	n.a.	**<0.0001 (+)**	n.a.	n.a.	n.a.	–

^a^ Cells show the results of 2 x 2 contingency table analysis; values in bold type are the p-values for pairs of parasites that occurred within chimpanzee hosts, i.e. within the same faecal samples, more (+) or less (-) often than expected by chance (p <0.05, with Holm–Bonferroni adjustment);

n.a. = no association (p>0.05)

### Leaf swallowing and *Oesophagostomum* infection

**Fig 6:**
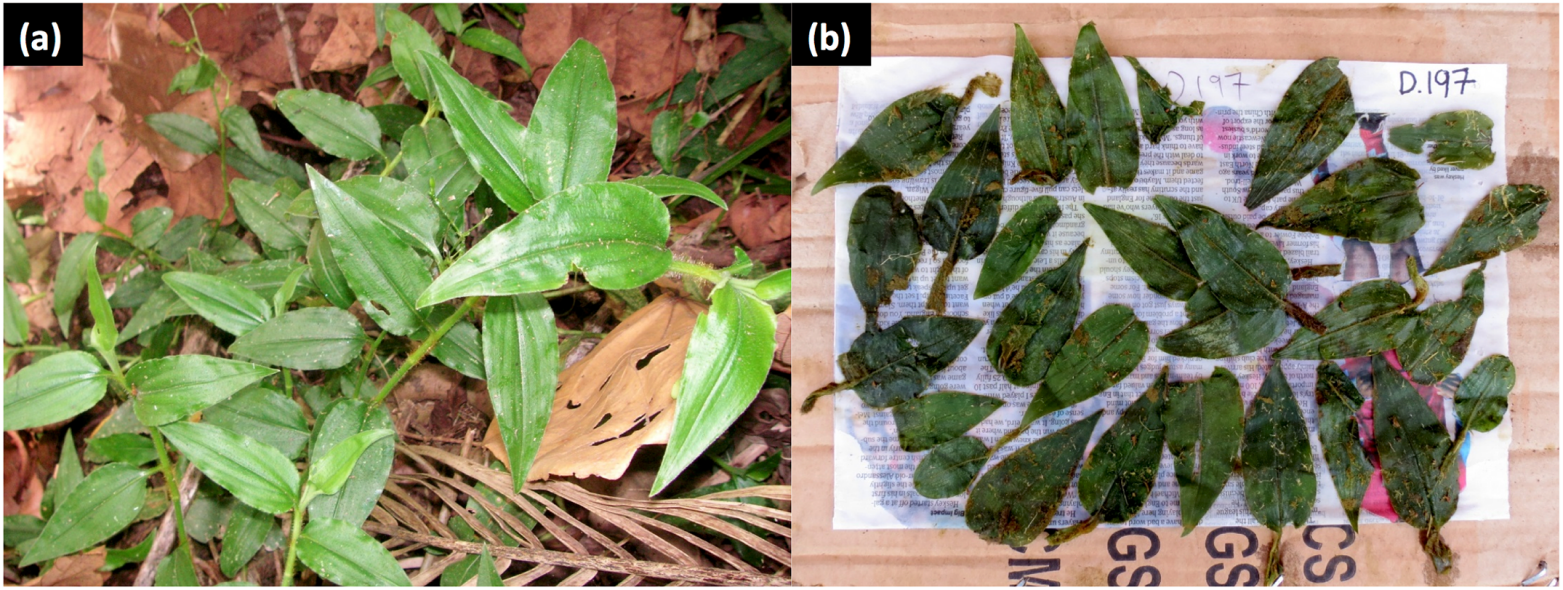
Chimpanzees in Bulindi commonly swallow whole bristly leaves of *Aneilema nyasense* C.B. Clarke (Commelinaceae). **(a)** *A*. *nyasense* growing in riverine forest in Bulindi. **(b)** 30 undigested and unchewed leaves of *A*. *nyasense* recovered from one chimpanzee faecal specimen; the leaves are passed in faeces folded one or more times, but have been unfolded manually here for identification. No adult worms were observed together with the leaves in this faecal sample.

Of the 406 faeces inspected visually, 11.8% (n = 48) contained folded unchewed leaves. The mean number of individual leaves distinguishable in faeces was 8.5 ± 10.6 SD (range: 1–46). In 96% of the cases (n = 46), the leaves were of the herb *Aneilema nyasense* (Fig 6). One faeces contained whole leaves of *Lantana camara*, an exotic invasive shrub; another contained an unidentified hispid grass blade, while three faeces contained a mixed use of *A*. *nyasense* with *L*. *camara* leaves (once) and *A*. *nyasense* with *Desmodium velutinum* leaves (twice) (S1 Data File).

**Fig 7:**
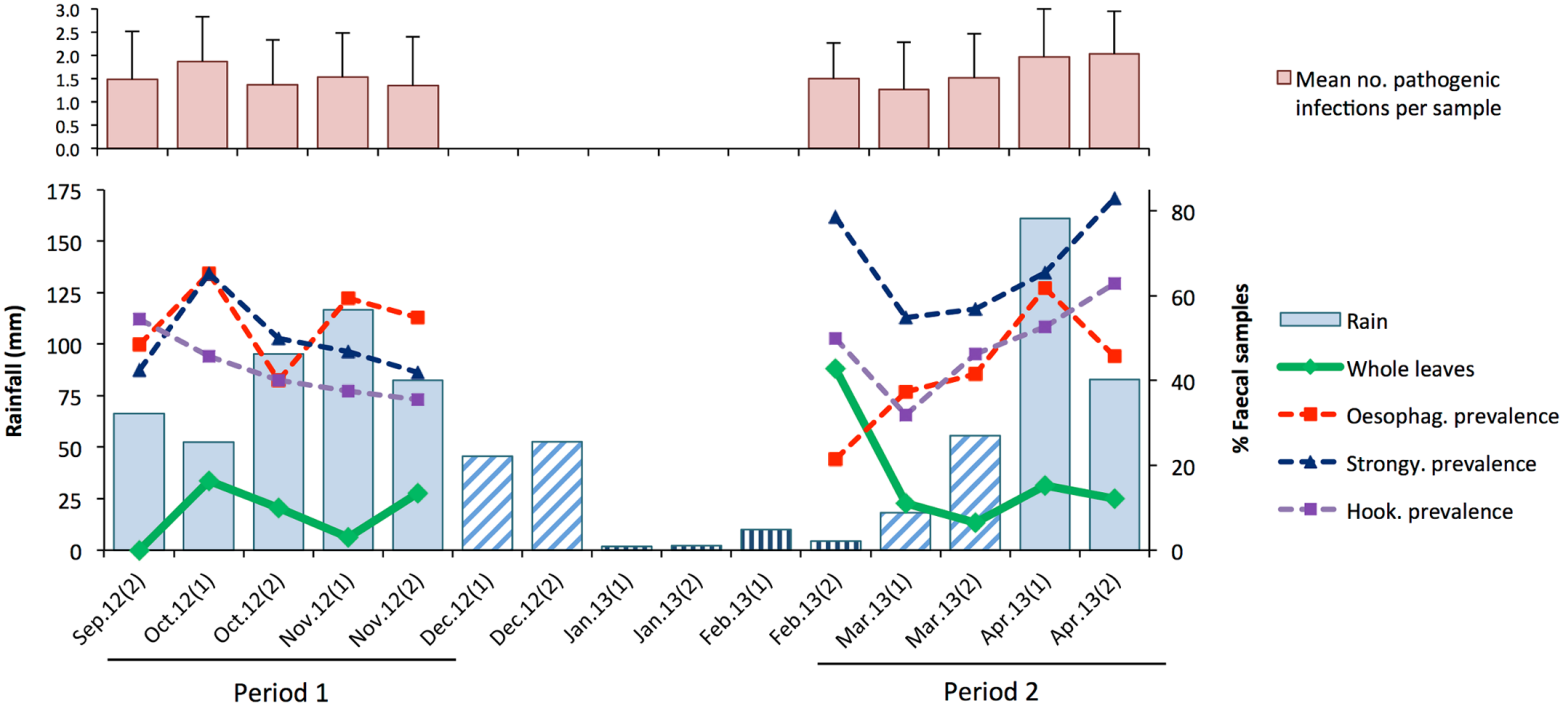
Temporal variation in leaf swallowing in relation to parasite infections and rainfall during the two survey periods. **Top**: bars show the biweekly mean (± SD) number of infections by different parasites with pathogenic potential, determined by coproscopy (see text). **Bottom**: Lines show the % biweekly faeces containing wholly swallowed leaves and the biweekly prevalence in samples of potentially pathogenic nematodes (*Oesophagostomum* sp., *Strongyloides* sp. and hookworm); bars show biweekly rainfall. The patterning of rainfall bars indicate whether biweekly periods were in months classified as wet (solid), transient (hatched) or dry (vertical lines); see Methods. The number of faecal samples inspected per biweekly period (microscopically; macroscopically): *Period 1* –Sep.2 (33; 28), Oct.1 (46; 43), Oct.2 (30; 30), Nov.1 (32; 33), Nov.2 (31; 30); *Period 2* –Feb.2 (14; 21), Mar.1 (91; 81), Mar.2 (65; 61), Apr.1 (55; 46), Apr.2 (35; 33).

Whole leaves occurred in faeces in nine of the ten biweekly periods (the exception being late September), in months classified as wet, dry and transient (Fig 7). The proportion of biweekly faeces containing whole leaves varied widely, ranging from 0–43%, with the highest proportion occurring in February, the end of a two month dry period. Fig 7 also shows the mean number of infections per sample (from coproscopy) with known or potential pathogenicity (i.e. *Balantidium coli*, *Giardia intestinalis*, *Bertiella* sp., hookworm, *Oesophagostomum* sp. and *Strongyloides* sp.) in each biweekly period. The mean number varied between 1.3 and 2.0 and showed no discernible fluctuation associated with changes in frequency of leaf swallowing or rainfall (Fig 7).

**Fig 8:**
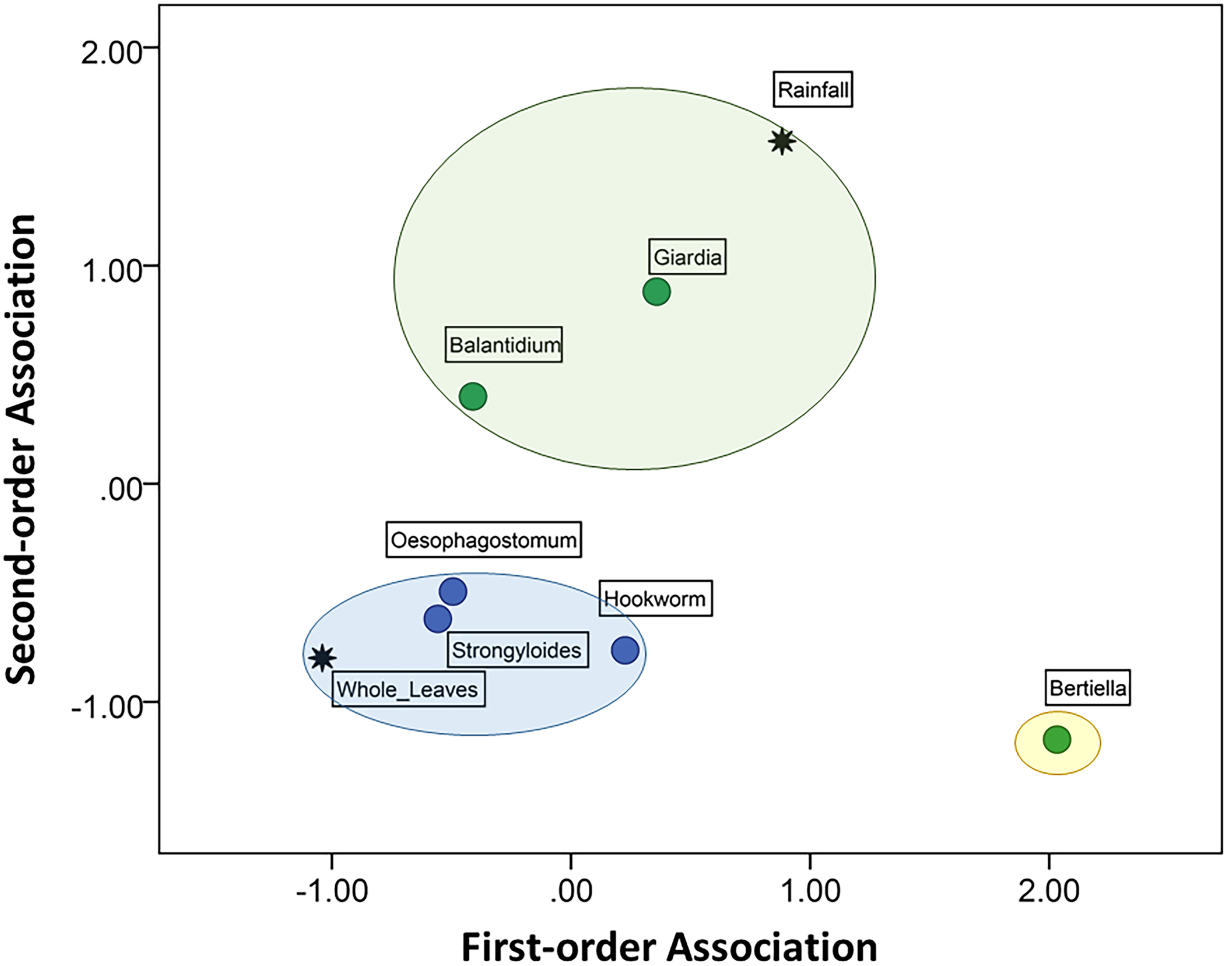
Multidimensional scaling (MDS) model of independent associations among whole leaf swallowing, rainfall, and six parasites with known or likely pathogenicity. The first-order dimension represents a stronger association than the second-order dimension. The closer two or more variables are, the more likely they covary independently of other points on the map. Colours differentiate groups of variables that show some degree of association in relation to other variables in the map.

The third MDS model revealed the independent associations among the frequency of whole leaves in faeces in biweekly periods and prevalence of potentially pathogenic parasites. (The matrix of optimal scaled distances is shown in Supporting Information S3 Table). Prevalence of each of the three nematodes–*Oesophagostomum* sp., *Strongyloides* sp., and hookworm—showed an association with temporal increases in leaf swallowing (stress = 0.058; R^2^ = 0.981; Fig 8), which was independent of rainfall as well as prevalence of other potentially pathogenic infections. Among nematodes, *Oesophagostomum* and *Strongyloides* had higher overall associations with whole leaves relative to hookworm.

Concerning macroscopic parasites, adult *Oesophagostomum stephanostomum* were found in 10 faeces (2.5% faeces examined macroscopically; Table 1). In four of these, ≥1 adult worms were found together with whole leaves. For example, one faeces collected in April 2013 (the wettest month in the survey, and when 6 of the 10 faeces with adult worms were collected) contained seven adult worms and 23 whole leaves of *Aneilema nyasense* (S1 Data File). Whole leaves and adult *O*. *stephanostomum* occurred together in faeces more often than expected (Fisher’s exact test, p = 0.021). Of three faeces containing a *Bertiella* sp. proglottid (0.7% faeces), one also had a whole *A*. *nyasense* leaf.

## Discussion

This study used coproscopy and molecular analyses to examine intestinal helminths and protozoa of wild chimpanzees inhabiting degraded forest amid farmland and villages in Bulindi. The number of distinct taxa detected microscopically (n = 16) was relatively high compared to most previous surveys of chimpanzee parasites, e.g. Assirik, n = 6 [42]; Lopé, n = 11 [33]; Mahale, n = 9 [39]; Kanyawara, Kibale, n = 15 [35]; Gombe, n = 10 [89]; Kanyawara, Kibale, n = 12 [36]; Ngogo, Kibale, n = 12 [37]; Gombe, n = 8 [90]; Rubondo, n = 12 [38]; Fongoli, n = 12 [43]; Mahale, n = 9 [41]; Cantanhez, n = 13 [30]; Budongo, n = 13 [46]; Ugalla, n = 8 [44]; Assirik, n = 11 [45], though it was lower than reported in a recent survey at Gombe (n = 17 [40]). However, variation in parasite diversity among sites and studies may be influenced as much by differences in sample size, survey duration and microscopic method(s) used, as actual differences in parasite faunas.

Parasites most frequently detected in chimpanzee faeces at Bulindi are also commonly found in populations elsewhere [56]. For example, *Troglodytella abrassarti* (80% faecal samples) is a non-pathogenic symbiont of wild apes thought to aid hindgut fermentation [47,48]. A second, less-frequently detected ciliate in our survey, *Troglocorys cava*, may provide similar digestive benefits [91]. Nematodes of the genera *Strongyloides*, *Oesophagostomum*, and *Necator* commonly parasitize wild apes (except in dry savanna habitats where conditions are less conducive to survival and transmission of parasites with free-living stages [44]). At Bulindi, their prevalence was relatively high (44–58% samples), although comparable, or higher, prevalence has been reported in some other East African chimpanzee populations, e.g. Gombe [40], Mahale [39,41,56], and Budongo [46,56]. The Bulindi chimpanzees were apparently devoid of certain nematodes reportedly found in some other populations of wild chimpanzees, i.e. whipworms, pinworms and ascarids [29,34,38,40,43,46,56].

Nevertheless, as noted above, cross-site comparisons are hindered by differences in diagnostic techniques used [35,56]. In general, prevalence and within-host richness (multiple infections) increases when >1 microscopic technique is used [36,40,92], and when individual infection rates are calculated from repeated sampling of known individuals, because samples from infected individuals do not always test positive [39]. Here, it was only possible to examine the full set of (anonymous) samples by simple sedimentation. However, 50 samples were examined initially by formalin–ether sedimentation as well. The mean number of parasite taxa detected in these 50 samples was 4.2 compared to 3.4 by simple sedimentation alone, which captured on average 79% of taxa detected with both methods. Further, samples that were positive for *Oesophagostomum*, *Strongyloides* or *Necator* by culture were sometimes negative for the same taxa by microscopy. Similarly, faeces containing adult *Oesophagostomum stephanostomum* did not always yield eggs, while none of three faeces with proglottids were positive for *Bertiella* eggs. Consequently, species richness in samples and prevalence was underestimated. The superior sensitivity of PCR-based methods for detecting parasites of primates has been demonstrated in several studies, e.g. *Blastocystis* sp. [93], *Entamoeba* spp. [88] and *Oesophagostomum* spp. [16,94].

DNA sequence analysis enabled more precise identification of helminths than was possible by microscopy. Molecular features of strongyle and rhabditoid nematodes in Bulindi chimpanzees are reported elsewhere (Table 2). An additional trichostrongyloid raised from culture aligned closest to the genus *Libyostrongylus* by similarity analysis. However, morphological studies of adults are also required to identify this nematode conclusively; thus we assign it to ‘Trichostrongyloidea fam. gen. sp.’ for the time being. This nematode was not identified by microscopy. However, trichostrongyle eggs are similar to those of *Oesophagostomum* sp. Thus, a small proportion of trichostrongyle eggs may have been misassigned to *Oesophagsotomum* sp. in our sample. If so, it is unclear if these are conspecific with the trichostrongyloid identified by culture. Notably, a related trichostrongyloid, *Paralibyostrongylus kalinae*, was described morphologically from *Gorilla beringei* in Uganda [95].

ITS1 sequences of the proglottids aligned most closely with those of *Bertiella studeri* and *B*. *satyri* (by FASTA and BLAST analysis, respectively), while ITS2 sequences were closest to ‘*Bertiella* sp.’ Thus, species-level identification could not be made with confidence. Doležalova et al. [96] reported a high variability of *Bertiella* isolates from human and non-human primates with little obvious geographical or host-related patterns, calling for revision of this cestode group using molecular and morphological data. A previous morphological examination of a larger sample of proglottids from Bulindi suggested a second cestode, tentatively identified as *Raillietina* sp., also infected the chimpanzees [54]. We found no evidence of this second putative species in the present study.

Where climatic conditions are conducive, infections of soil-transmitted helminths including *Oesophagostomum*, *Strongyloides* and *Necator* increase after the onset of wet months ([32,36,39,40, 56]; cf. [38,57]). At Bulindi, *Oesophagostomum* was detected in faecal samples at a higher frequency in wet compared to dry or transient months (Table 3). However, multidimensional scaling (MDS) analysis revealed that an increase in *Oesophagostomum* infection was most clearly associated with periods of low temperature rather than rainfall. Nevertheless, the frequency of positive samples rose steadily after the January–February dry season (Fig 3). Thus, there was a general pattern for *Oesophagostomum* prevalence to increase with increasing rainfall, which accords with the expected increase in reinfection preceding the onset of rains reported at Mahale [39]. Furthermore, the seasonal distribution of rainfall is dissimilar enough between Bulindi and Mahale to expect variation in the overall patterns observed. Univariate analysis suggested no difference in prevalence of *Strongyloides* sp. between wet and dry–transient months (Table 3). However, an association between periods of increased *Stronglyoides* infection and lower temperature and rainfall was revealed by MDS (Fig 4).

Protists multiply within hosts and once established can persist for long periods, regardless of climatic conditions. Even so, several protists (*Blastocystis* sp., *Entamoeba coli* and *Troglocorys cava*) and *Probstmayria gombensis*, a non-pathogenic nematode, were detected most frequently in samples in the transition from the dry season (February) to the onset of heavy rains in April (Fig 3). MDS revealed these infections were associated with climate in different ways. For example, *Blastocystis* and *Probstmayria gombensis* were both more commonly detected in periods of high temperature. But whereas *Blastocystis* was also associated with higher rainfall and humidity, the opposite was true of *Probstmayria*. Some association patterns revealed by MDS defy obvious explanation (e.g. the temporal separation of *Troglodytella abrassarti* and *Entamoeba* sp. from other protists in Fig 5). Interpreting seasonal infection patterns including temporal associations among parasites is problematic without a firm understanding of the ecology of the individual organisms and close monitoring and evaluation of microclimatic and annual fluctuation in climatic conditions. Variation among parasite taxa and between sites will reflect climatic and ecological influences on the lifecycle of parasites, as well as potential changes in diet and ranging of chimpanzee hosts. Longer-term monitoring—including more sampling in the dry season than was possible here—is inevitably needed to better evaluate causes of temporal variation in infections within and among taxa at Bulindi.

Several pairs of parasites occurred together within chimpanzee hosts more or less often than expected by chance. Experimental and field studies in other species confirm that parasite associations (positive or negative) arise from complex interactions mediated by host immune responses and competition among co-infecting parasites (for a review, see [59]). For example, in this study the commensal ciliate *Troglodytella abrassarti* was strongly negatively associated with both *Balantidium coli* and *Oesophagostomum* sp. (though these pathogenic taxa were not themselves associated). Possibly, infection with *Balantidium* or *Oesophagostomum* makes the large intestine less suitable for *Troglodytella* (i.e. interference competition). Conversely, the positive association of *Troglodytella* with *Strongyloides* sp. within chimpanzee hosts is difficult to explain because both inhabit different parts of the intestine. For other pairs, similar climatic influences on parasite life cycles (e.g. soil-transmitted nematodes) are probably also drivers of these interactions. Interestingly, while hookworm and *Oesophagostomum* were positively associated within chimpanzee hosts, these nematode infections did not cluster together temporally (Fig 5). As noted above, however, some infections were likely underestimated by microscopic examination. Thus, these results should be regarded as indicating general patterns only.

Almost 12% of faeces contained unchewed whole leaves resulting from leaf swallowing. This is consistent with our earlier study [54], which reported a higher occurrence of whole unchewed leaves in chimpanzee faeces in Bulindi (10.4% faeces), compared to other sites (≤4% faeces). In both of our studies, separated by four years, Bulindi chimpanzees mainly swallowed leaves of *Aneilema nyasense*. Fruth et al. [97] observed that since bonobos (*Pan paniscus*) and chimpanzees usually swallow leaves early in the morning and pass them in faeces later in the day, analysis of faeces deposited throughout the day should reveal a higher occurrence of unchewed leaves compared to faeces deposited early in the morning. At Bulindi, however, this does not explain the higher proportion of faeces with whole leaves found in this study, since faeces were collected in the first half of the day only.

Leaf swallowing at Bulindi occurred in every biweekly period of this study except one (late September), suggesting no strong seasonality, which may be related to the fact that *Oesophagostomum* infections were also noted in every biweekly period (Fig 7), albeit with some variation as discussed above. In fact, temporal variation in the frequency of leaf swallowing was independently associated with all three pathogenic nematodes–*Strongyloides* sp. and hookworm (presumably *Necator* sp.), as well as *Oesophagostomum* sp.–with *Strongyloides* and *Oesophagostomum* showing strongest associations (Fig 8). However, since we did not measure infection intensity (eggs per gram faeces) we could not differentiate between severe or mild infections, and faecal samples were not individually identified. Thus, we were unable to draw conclusions about specific relationships between leaf swallowing behaviour and levels of *Oesophagostomum* infection or other pathogenic nematodes in individuals. Understanding this relationship better will require tracking infection levels and leaf swallowing behaviour in individual chimpanzees over time.

Nevertheless, as in the earlier study [54], whole leaves occurred together with adult *Oesophagostomum stephanostomum* in faeces more often than expected by chance, indicating a link between leaf swallowing and expulsion of adult nodular worms at Bulindi (as also demonstrated at Mahale: [39,52,53]). Wrangham [55] and Huffman et al. [56] also found associations between leaf swallowing and *Bertiella* sp. infection in Ugandan chimpanzees at Kibale and Budongo, respectively, because the leaves presumably stimulate gravid segment apolysis, which is a natural part of the cestode’s life cycle. In this study, a low prevalence of *Bertiella* infection was suggested both by microscopy and the rare incidence of proglottids in faeces. Proglottids appeared more frequently in the earlier study (2.5% faeces compared to 0.7% in this study), but were not associated with whole leaves [54]. However, the lack of an association at Bulindi could be due to a low level of detection. Cyclophyllid cestodes do not oviposit eggs inside the host’s intestine. Rather, the eggs found in faeces are shed by gravid segments [98]. Thus, actual prevalence may be much higher than suggested by faecal examination. At Mahale too, *Bertiella* proglottids or eggs were extremely rare (0.7% of 1769 faeces examined macroscopically or microscopically), and only once were leaves and proglottids found together, along with an adult *Oesophagostomum stephanostomum* worm [56]. Currently, however, our data does not support the hypothesis that leaf swallowing at Bulindi is related to the control of tapeworm infection [54], or that of *Strongyloides* and hookworm nematodes.

The high frequency of leaf swallowing at Bulindi remains difficult to explain by climatic factors and frequency of infections at the community level, without data on intensity of individuals’ infections. Possibly, it is a generalised response to gastrointestinal discomfort caused by multiple infections and relatively constant levels of infection (Fig 7), while still functioning to reduce *Oesophagostomum* worm burdens through the expulsion of adult worms. Additional unknown factors may be involved, warranting further investigation. An understanding of the reasons behind the unusually high frequency of leaf swallowing at this site, may lead to a broader understanding of the behaviour in great apes and indeed other species that also exhibit this behaviour [99–101].

### Pathogenicity and zoonotic potential

Many parasites demonstrated in this survey are non-pathogenic (e.g. the commensal ciliates and other protists like *Chilomastix mesnili*, *Entamoeba coli* and *Iodamoeba buetschlii*). Others have strong potential pathogenicity, especially the rhabditoid and strongyle nematodes, and *Balantidium coli* and *Giardia intestinalis*. However, their potential to cause disease in wild chimpanzees is in most cases inferred on the basis that they (or closely related taxa) cause disease in humans, captive apes or other primates [26,102]. With the exception of *Oesophagostomum* infection, for which severe clinical symptoms (e.g. weight loss, lethargy, enteritis, diarrhea) have been documented at Mahale [39] and Gombe [49] the rarity of overt clinical signs of disease linked to specific intestinal parasites in wild apes suggests many species infections are tolerated. At the time of this study, chimpanzees in Bulindi were insufficiently habituated to observe such clinical signs. More recently, however, intestinal lesions consistent with multinodular oesophagostomiasis (cf. [49,103]) were noted in a well-nourished adult female at Bulindi that died after colliding with a vehicle [104]. While enteritis was also noted at necropsy, no overt behavioural indicators of sickness were observed in this female in the month prior to death. This might suggest that in most cases, *Oesophagostomum* spp. infections are tolerated and/or mediated by self-medicative behaviours like leaf swallowing. Nevertheless, certain gastrointestinal infections detected in this survey including *Oesophagostomum* spp. might cause chronic low-intensity infections that reduce host fitness, in the absence of overt clinical symptoms.

All parasites identified to genus or species in our sample have been reported in wild chimpanzees living in less disturbed environments and with less contact with local people and livestock [56]. Aside from *Giardia intestinalis*, which could have resulted from contact with human or livestock faeces, but which was detected at a low prevalence, we did not identify any infections attributable to the degraded, human-dominated habitat at Bulindi *per se*. Even so, several disease-causing parasites infecting the chimpanzees are potentially transmissible among great apes and humans, especially soil-transmitted nematodes and species of pathogenic protozoa that spread via faecal–oral transmission. Molecular analysis of parasites from both apes and humans sharing environments—and domestic animals where applicable—are required to evaluate host specificity and establish if zoonotic exchange occurs (e.g. [16,17,32,77,78,105,106]). The remarkable close contact between wild chimpanzees and villagers in Bulindi and throughout the wider region [61–66] underscores the importance of further studies of pathogens harboured by coexisting apes, local humans and livestock—including bacterial and viral pathogens—to more fully evaluate threats to chimpanzee and public health, and to aid development of strategies to reduce risks.

## Supporting information

S1 Data File Spreadsheet containing raw data on which all statistical analyses in this paper are based on. (XLSX)

S1 Table Matrix of stimulus coordinates for the MDS model of independent associations among parasites and climate. (PDF)

S2 Table The matrix of stimulus coordinates for the MDS model of independent associations among common parasites. (PDF)

S3 Table The matrix of stimulus coordinates for the MDS model of independent associations among leaf swallowing and pathogenic parasites. (PDF)
