# Supplementary material for: Gastrointestinal parasite infections and self-medication in wild chimpanzees surviving in degraded forest fragments within an agricultural landscape mosaic in Uganda
Source: PLoS One. 2017 Jul 10;12(7):e0180431. doi: 10.1371/journal.pone.0180431 (PMC5503243; doi:10.1371/journal.pone.0180431)
Supplement: S1 Table — (PDF) [file pone.0180431.s002.pdf]

Supporting Information

S1 Table. Matrix of stimulus coordinates for the MDS model of independent associations among parasites and climate.[1]

	Climate variables				Parasites					
	Rainfall	Humidity	Max. Temp	Min.Temp	*Troglocorys*	*Entamoeba coli*	*Blastocystis*	*Strongyloides*	*Oesophagostomum*	*Probstmayria*
Rainfall	0									
Humidity	0.93	0								
Max. Temp	3.918	4.621	0							
Min. Temp	1.5	1.713	3.102	0						
Troglocorys	3.102	3.695	1.479	1.713	0					
Entamoeba coli	2.14	2.557	2.523	0.93	1.713	0				
Blastocystis	1.479	2.291	2.523	1.47	2.221	1.479	0			
Strongyloides	2.557	3.048	1.713	0.93	0.688	0.93	1.563	0		
Oesophagostomum	1.999	2.221	2.557	0.688	1.479	0.93	1.713	0.688	0	
Probstmayria	2.523	3.211	1.479	1.713	0.93	1.5	0.93	0.907	1.563	0

[1] The table shows the optimally scaled data (disparities) for the MDS analysis of independent associations among biweekly climatic conditions and parasite prevalence (frequency of positive samples). Disparities represent the strength of association among the variables included in the model. Larger disparities are equivalent of a weak association. Only parasite taxa that showed a significant difference in prevalence between survey periods were selected for the model (see Table 1).
