# Supplementary material for: Gastrointestinal parasite infections and self-medication in wild chimpanzees surviving in degraded forest fragments within an agricultural landscape mosaic in Uganda
Source: PLoS One. 2017 Jul 10;12(7):e0180431. doi: 10.1371/journal.pone.0180431 (PMC5503243; doi:10.1371/journal.pone.0180431)
Supplement: S2 Table — (PDF) [file pone.0180431.s003.pdf]

Supporting Information

S2 Table. The matrix of stimulus coordinates for the MDS model of independent associations among common parasites.[1]

	Balintidium	*Blastocystis*	*Entamoeba coli*	*Entamoeba* sp.	Hookworm	*Oesophagostomum*	*Probstmayria*	*Strongyloides*	*Troglocorys*	*Troglodytella*
Balintidium coli	0									
Blastocystis sp.	2.616	0								
Entamoeba coli	2.486	2.252	0							
Entamoeba sp.	2.554	3.345	2.751	0						
Hookworm	2.165	2.23	1.444	2.042	0					
Oesophagostomum sp.	1.401	2.693	2.186	1.822	1.333	0				
Probstmayria gombensis	3.034	2.028	2.498	2.158	1.982	2.563	0			
Strongyloides sp.	2.111	2.539	1.994	1.59	0.989	1.366	1.713	0		
Troglocorys cava	2.969	3.24	3.023	0.992	2.309	2.287	1.744	1.667	0	
Troglodytella abrassarti	2.403	2.997	1.947	1.123	1.165	1.489	2.049	0.905	1.565	0

[1] The table shows the optimally scaled data (disparities) for the MDS analysis of independent associations among biweekly parasite prevalence (frequency of positive samples). Disparities represent the strength of association among the variables included in the model. Larger disparities are equivalent of a weak association. Only parasites detected in >5% of faecal samples were included in the model.
