# Supplementary material for: Gastrointestinal parasite infections and self-medication in wild chimpanzees surviving in degraded forest fragments within an agricultural landscape mosaic in Uganda
Source: PLoS One. 2017 Jul 10;12(7):e0180431. doi: 10.1371/journal.pone.0180431 (PMC5503243; doi:10.1371/journal.pone.0180431)
Supplement: S3 Table — (PDF) [file pone.0180431.s004.pdf]

Supporting Information

S3 Table. The matrix of stimulus coordinates for the MDS model of independent associations among whole leaf swallowing and pathogenic parasites.[1]

	Rainfall	*Oesophagostomum*	Hookworm	*Strongyloides*	*Giardia*	*Balantidium*	*Bertiella*	Whole Leaves
Rainfall	0							
Oesophagostomum sp.	2.123	0						
Hookworm	2.295	0.935	0					
Strongyloides sp.	2.539	0.959	0.694	0				
Giardia intestinalis	1.486	1.851	1.79	1.965	0			
Balantidium coli	1.786	0.983	1.519	1.481	1.693	0		
Bertiella sp.	2.544	2.541	1.98	2.34	2.469	2.85	0	
Whole Leaves	2.94	2.896	2.857	2.647	2.359	2.7	2.9	0

[1] The table shows the optimally scaled data (disparities) for the MDS analysis of independent associations among the biweekly frequency of whole swallowed leaves in faeces, rainfall, and parasite prevalence. Disparities represent the strength of association among the variables included in the model. Larger disparities are equivalent of a weak association. Only parasites with known or likely pathogenicity were included in the model.
